# Psychosocial burden of discrimination based on sexual orientation in university students—A cross-sectional analysis

**DOI:** 10.3389/fpubh.2026.1799079

**Published:** 2026-05-05

**Authors:** Konrad Jakob Endres, Aneliana da Silva Prado, Sarah-Lena Klemm, Juliane Hug, Elisabeth Kohls, Christine Rummel-Kluge

**Affiliations:** 1 Department of Psychiatry and Psychotherapy, Medical Faculty, Leipzig University, Leipzig, Germany; 2 Federal Institute of Education, Science, and Technology of Parana, Curitiba, Brazil; 3 Department of Psychiatry and Psychotherapy, University Leipzig Medical Center, Leipzig, Germany

**Keywords:** discrimination, mental health, minority stress, sexual orientation, university students

## Abstract

**Introduction:**

Sexual minority individuals continue to face prejudice and discrimination within medical care and academic settings. This contributes to elevated stress levels, which increases their vulnerability to mental health issues like anxiety and depression. Studies examining the mental health, financial situation, loneliness, and help-seeking behavior of students who have experienced discrimination based on their sexual orientation remain scarce. Therefore, this study aims to address this research gap by assessing mental health outcomes, loneliness, impairment status, income, and help-seeking behavior in a large cohort of university students in eastern Germany, comparing these variables between participants who experienced discrimination due to sexual orientation and those who did not.

**Methods:**

An online survey was conducted to assess university students' mental health, as well as to identify relevant risk and protective factors. The questionnaire included questions on various forms of discrimination experienced by participants, including discrimination based on sexual orientation. The final sample comprised 2,625 students, of which 2,569 answered the questionnaire for discrimination experiences and were included in this study. Sociodemographic variables, income, mental health outcomes (assessed via PHQ-9, GAD7, EDE-Q8, and AUDIT-C), help-seeking behavior, impairment status, and loneliness (measured with UCLA-3) were used to examine group differences between participants reporting experiences of discrimination based on sexual orientation and those without discrimination based on sexual orientation.

**Results:**

Results indicated that participants reporting discrimination based on sexual orientation had a significantly higher prevalence of diagnosed mental illness, impairment affecting study, and suicidal thoughts, as well as a higher mean PHQ-9 and GAD-7 score compared to the students without experiences of discrimination based on sexual orientation. Students facing discrimination based on sexual orientation reported more frequent impairments affecting their studies. No significant differences between the groups were observed in hazardous alcohol use (AUDIT-C), eating disorder symptoms (EDE-Q8), loneliness (UCLA-3) and monthly income.

**Discussion:**

The findings highlight the association between discrimination based on sexual orientation and poorer mental health among sexual minority university students. Moreover, the results emphasize the need for targeted mental health services and more inclusive support structures for sexual minority students at university.

## 1 Introduction

Sexual minority individuals continue to face discrimination and systemic inequities worldwide, manifesting in legal barriers, social exclusion, and significant health disparities (1). As a result, sexual minority individuals frequently encounter considerable challenges in academic (2) and healthcare environments due to ongoing stigma and structural inequities (3).

A growing body of literature suggests that sexual minority university students are more likely to develop a mental health disorder in comparison to cisgender/heterosexual students (4, 5). For example, sexual minority university students were found to present worse depressive symptoms, higher levels of perceived stress and considered themselves less attractive (6). Another study indicated that sexual minority students were more likely to report drug use and more alcohol and other drug use (7). A meta-analysis including over 19 studies and a total of 122,955 participants found that sexual minority youth have significantly higher rates of suicidality, with an odds ratio (OR) of 2.92 compared to heterosexual youth (8). The elevated prevalence of suicidality among sexual minority youth is a significant concern, especially as evidence indicates that the majority of sexual minority university students experiencing suicidal ideation do not seek help (9). As such, these findings highlight persistent mental health disparities among sexual minority university students, underlining the importance of examining the underlying psychosocial and contextual factors that may contribute to their increased vulnerability.

Building on this evidence, research has also explored specific mechanisms linking discrimination to adverse health outcomes among sexual minority individuals. In a sexual minority sample with over 440 participants in Turkey, the total number of heterosexist experiences predicted disturbed eating behavior (10). Heterosexist experiences refers to instances of prejudice, discrimination, or negative treatment that sexual minority individuals face because heterosexuality is considered the norm or superior, and non-heterosexual orientations are devalued and seen as inferior (11). Taken together, these findings have major public health relevance, as recent surveys show that 22.7% of Generation Z (born 1997–2012) identify as LGBTQ+, a proportion that is significantly higher than in previous generations (12).

Despite affecting a substantial and growing proportion of university students, financial hardship among sexual minority students has received little scientific attention. Family rejection, which has been identified as a risk factor for several adverse health outcomes such as suicidality, substance abuse, and depression (13), could also have substantial financial implications. When young adults experience rejection or reduced support from their families due to their sexual orientation, they may lose access to financial resources that typically help students manage living and study expenses. Given prior findings showing that financial hardship is associated with poorer mental health among sexual minority men (14), examining the monthly income of sexual minority university students provides a valuable opportunity to explore how family rejection might translate into financial strain. This is particularly important, as sexual minority students may encounter unique financial and social challenges compared to their heterosexual peers (15).

At the same time, it is crucial to understand not only the financial circumstances of sexual minority students but also how they engage with available mental health services. Sexual minority university students were shown to seek help at mental health services more frequently than heterosexual students (16). This increased need for support is largely due to the higher prevalence of mental health issues among sexual minority individuals, which can be attributed to experiences of discrimination, stigma, and social rejection (17). While several international studies have examined healthcare preferences among sexual minority individuals, focussing on provider choice and perceived safety in health care settings (18, 19), there remains a lack of research regarding sexual minority individuals' preferences for health care providers (e.g., general practitioners, psychotherapists, and psychiatrists) and mental health treatment settings (e.g., inpatient vs. outpatient care) specifically in Germany.

The poorer physical and mental health of sexual minority individuals has multifactorial causes. However, elevated stress due to their minority status is particularly significant in this case. Discriminatory actions expose individuals belonging to a minority group, in this case, a sexual minority, to substantial psychosocial stress, which in turn increases their vulnerability to various (mental) health conditions (20). In a recent study, sexual minority adults reported having lower levels of perceived social support and were more likely to experience loneliness than their heterosexual counterparts (21). Considering the negative impact that loneliness can have on both mental and physical health (22), loneliness was shown to mediate the relationship between minority stressors and psychological distress in a sample of 719 sexual minority adults (23).

Besides the association between minority status and mental health problems, there is an increasing number of studies that have demonstrated the negative impact of discrimination based on sexual orientation on mental health (24, 25), which makes experiences of discrimination due to sexual orientation an important factor to consider in scientific mental health research.

However, research investigating the mental health, impairment status, income differences, and help seeking behavior of university students who have experienced discrimination based on sexual orientation remains scarce. Therefore, this study aims to address this research gap by assessing mental health outcomes, loneliness, impairment status, income, and help-seeking behavior in a large cohort of university students in eastern Germany, comparing these variables between participants who experienced discrimination due to sexual orientation and those who did not.

To this end, the research questions were as follows: (1) whether students who reported discrimination based on their sexual orientation experienced poorer mental health and greater loneliness compared to those who did not report this kind of discrimination; (2) whether these students have had a higher prevalence of impairments that made studying more difficult; (3) whether they were more likely to face financial hardships and have a lower monthly income; and (4) whether their help-seeking behavior differed from that of students who did not report discrimination based on sexual orientation.

To our knowledge, no study has simultaneously examined all these factors (mental health outcomes, loneliness, impairment status, income, and help-seeking behavior) in relation to experiences of discrimination based on sexual orientation within a large student population. This comprehensive approach allows for a more integrated understanding of how discrimination based on sexual orientation influences multiple aspects of mental wellbeing and functioning.

## 2 Methods

### 2.1 Participants and procedures

This cross-sectional study was conducted in May-June 2025. A participation link to the study was distributed to all students at the University of Leipzig per email. All questionnaires in the survey were anonymous, self-administered, and based on self-report. Participants could not be identified or followed longitudinally, and confidentiality was fully ensured throughout the data collection process. The survey was conducted using Unipark (EFS Survey; Questback GmbH, Cologne, Germany). A total of *N* = 3,098 university students began the online survey, with *n* = 2,631 completing it up to the Patient Health Questionnaire (PHQ-9), which included the primary outcome measures of the study. Among the remaining respondents, *n* = 5 participants reported not answering the questionnaire seriously in a validity check and were therefore excluded from the analysis. Additionally, one case (*n* = 1) with implausible data was removed following a manual review of descriptive statistics. This resulted in a sample size of *n* = 2,625 participants. Of these, *N* = 2,569 participants answered the question regarding discrimination based on sexual orientation and therefore comprised the final sample in this study.

### 2.2 Measures

The online survey utilized a series of standardized questionnaires as follows (see Table 1).

**Table 1:** Summary of measurement instruments used in the study.

Instrument	Items	Response scale	Score range	Clinical cut-off	References (ENG, GER)
PHQ-9 (Patient health questionnaire-9)	9	0–3 on Likert scale	0–27	≥10	(26, 59)
GAD-7 (Generalized Anxiety Disorder-7)	7	0–3 on Likert scale	0–21	≥ 10	(27, 60)
UCLA 3-Item Loneliness Scale	3	1–3 on Likert scale	3–9	n.a.	(29)
AUDIT-C (Alcohol Use Disorders Identification Test-Consumption)	3	0–4 on Likert scale	0–12	≥5 in men, ≥4 in women	(61, 62)
EDE-Q8 (Eating Disorder Examination Questionnaire-8)	8	0–6 on Likert scale	Mean score, 0–6	n.a.	(30, 63)

#### 2.2.1 Sociodemographic information

Several sociodemographic variables were collected via self-report to characterize the study sample. Gender was assessed using three categories: female, male, and diverse gender identities. Age and body mass index (BMI) were recorded as continuous variables. BMI was calculated from self-reported height and weight after filtering for plausible values (heights between 120 and 210 cm, weights between 30 and 200 kg). The body mass index (BMI) was calculated by dividing body weight in kilograms by the square of height in meters.

Relationship status was categorized into married, in a committed relationship, single, or divorced. Residential status distinguished between living alone or with a partner/spouse/children/others. Migration history was assessed by asking participants about their or their family's migration background. Lastly, parental status was recorded as a binary variable indicating whether participants were parents or not. All variables were measured to describe the sample and to compare groups based on experiences of discrimination related to sexual orientation.

#### 2.2.2 Income assessment

Income was measured using a self-reported categorical question where participants selected their monthly net income range from predefined choices. The income categories included: no income, 0–499 €, 500–999 €, 1000–1499 €, 1500–1999 €, 2000–2999 €, 3000 € and more, and a “no answer” option.

#### 2.2.3 Mental health diagnosis

Participants were asked whether they had ever been diagnosed with a mental illness by simply indicating yes or no. Additionally, they had the option to specify what mental disorder they had been diagnosed with.

#### 2.2.4 PHQ-9

The PHQ-9 is a self-rating questionnaire with nine items designed to assess the prevalence and severity of depressive symptoms over the past 2 weeks (26). Each item reflects a DSM diagnostic criterion for major depressive disorder and is rated on a four-point Likert scale ranging from 0 (“not at all”) to 3 (“nearly every day”). Consequently, a higher total score indicates a greater severity of depressive symptoms. A score of 10 or above is used as clinical cut-off score, suggesting the presence of clinically relevant depressive symptoms. We additionally assessed the presence of suicidal thoughts based on PHQ-9 item 9, where responses of 1 (“*several days*”) or higher were coded as indicating suicidal thoughts, while a response of 0 (“*not at all*”) was interpreted as the absence of suicidal thoughts.

#### 2.2.5 GAD-7

The GAD-7 is a brief, self-administered questionnaire used to screen for and measure the severity of generalized anxiety disorder. It consists of seven items that ask respondents how often they have experienced common anxiety symptoms over the past 2 weeks. Scores range from 0 to 21, with higher values indicating greater levels of anxiety (27).

#### 2.2.6 AUDIT-C

The Alcohol Use Disorders Identification Test-Consumption (AUDIT-C) is a brief screening questionnaire consisting of three items that assess the frequency and quantity of alcohol consumption. Each item is rated on a scale from 0 (“never”) to 4 (“4 or more times a week”), resulting in a total score ranging from 0 to 12. Higher scores indicate increased alcohol use (28).

#### 2.2.7 UCLA 3-Item Loneliness Scale

Loneliness was assessed using an adapted version of the UCLA 3-Item Loneliness Scale (29). Participants were asked how often they experienced three feelings: lacking companionship, feeling left out, and feeling isolated from others. For each item, responses were given on a three-point scale (from 1 = “hardly ever” to 3 = “often”).

#### 2.2.8 EDE-Q8

The EDE-Q8 is a brief version of the Eating Disorder Examination Questionnaire, consisting of eight items that evaluated key behaviors and attitudes related to eating disorders (30). This short self-report measure assesses core dimensions such as restraint, eating concern, shape concern, and weight concern. The response options for the items ranged from 0 to 6. A sum score was calculated by adding the responses to all eight items, which was then divided by the number of items to obtain a mean score. Higher mean scores on the EDE-Q8 indicated greater severity or higher frequency of eating disorder symptoms.

#### 2.2.9 Assessment of discrimination experiences

To assess experiences of discrimination, participants were asked whether they had experienced discrimination in the past 2 years for any of the following reasons: ethnicity or origin, gender, gender identity (e.g., transgender or intersex), disability or chronic illness, old age, being too young, sexual orientation (operationalised through sexual identity e.g., gay, lesbian, and bisexual), low level of education, or low income. For each category, participants could respond with “Yes,” “No,” or “No answer.” An additional option allowed participants to indicate discrimination for another reason, which could be specified in a free-text field.

#### 2.2.10 Impairment and disability assessment

Students were asked if they had an impairment that made studying more difficult. Students who reported an impairment were asked to specify the type of impairment they experienced. The categories included psychological disorders, physical chronic illnesses, learning disabilities, mobility impairments, visual or hearing impairments, multiple severe impairments, and other types of impairments or illnesses.

#### 2.2.11 Assessment of help-seeking behavior

Help-seeking behavior for mental health problems was assessed with single-choice items, where participants were asked to indicate whether they had “*already sought help,”* “*sought help within the past year,”* or if the source was “*not applicable.”* Each item referred to a specific help-seeking source, including both formal and informal sources. For statistical analysis, responses were dichotomized by combining the categories “*already sought help*” and “*sought help within the past year*” into a single “*help-seeking*” group.

In addition to depressive symptoms (PHQ-9), we assessed anxiety (GAD-7), alcohol use (AUDIT-C), and eating disorder symptoms (EDE-Q8) to capture a broader spectrum of mental health and behavioral correlates. These measures were included given previous evidence linking minority stress and discrimination experiences to elevated anxiety (31), substance use (24), and disordered eating among sexual minority populations (32).

### 2.3 Statistical analyses

To examine group differences between students who reported discrimination based on sexual orientation and those who did not, a range of sociodemographic, mental health, financial, and impairment-related variables were analyzed.

Categorical variables, including gender, relationship status, residential status, migration history, parental status, presence and type of impairment, income categories, and help seeking behavior were compared using Chi-square tests of independence. Test statistics, degrees of freedom, exact *p*-values, and phi coefficients (φ) calculated for 2 × 2 tables (or Cramér's V (φ_*c*_) for larger contingency tables) were reported as measures of effect size. Effect size interpretation followed common guidelines, where values of 0.10 represent a small effect, 0.30 a medium effect, and 0.50 a large effect for both φ and φ_*c*_ (33).

Continuous variables such as age, body mass index (BMI), and questionnaire scores assessing mental health outcomes (PHQ-9, GAD-7, UCLA 3-Item Loneliness Scale, AUDIT-C, and EDE-Q8) were analyzed using Mann-Whitney U tests due to non-normal distributions or ordinal scaling of the data. The U statistic, exact *p*-values, and rank-biserial correlation coefficients (*r*) were reported to quantify effect sizes (33).

Effect sizes were interpreted according to conventional benchmarks to provide meaningful context to the statistical findings. Statistical significance was evaluated at the 0.05 alpha level. The statistical analyses were conducted using R version 4.5.0. The analysis scripts are available upon request.

To control for multiplicity and reduce the Type I error rate, the Bonferroni correction was applied, adjusting the original significance level of α = 0.05 to an adjusted α = 0.00208 (*n* = 24), ensuring a more conservative interpretation of our findings.

## 3 Results

### 3.1 Study sample characteristics

The study sample comprised a total of 2,569 students, among whom 10.59% (*n* = 272) reported having experienced discrimination based on sexual orientation in the past two years, while the remaining 89.41% (*n* = 2,297) reported no experiences of discrimination based on sexual orientation (see Table 2). Sociodemographic characteristics including age, BMI, relationship status, residential status, migration history, and parental status did not differ significantly between groups. However, in gender distribution a significant difference between the groups was observed (χ^2^ (2, 2569) = 109.41, *p* < .001, φ_*c*_ =.206), with a notably higher proportion of diverse gender identities among students experiencing discrimination based on their sexual orientation (15.44%) compared to those not experiencing this kind of discrimination (2.53%). Female representation was also significantly lower in the group experiencing discrimination based on sexual orientation (59.46% vs. 71.75%), while male proportions were similar across groups (15.73% vs. 25%).

**Table 2:** Sociodemographic information of the sample (*N* = 2,569).

Variable, *n* (%)	Students without discrimination based on sexual orientation (*n* = 2,297)	Students with discrimination based on sexual orientation (*n* = 272)	Test	*p*	Effect size
Gender, *n* (%)			*χ^2^*(2, 2569) = 109.41	< 0.001	*φ_*c*_* = 0.206
-Female	1,648 (71.75)	162 (59.46)			
-Male	591 (25.73)	68 (25)			
-Diverse	58 (2.53)	42 (15.44)			
Age, *M* (*SD*)	24 (4.65)	23.7 (3.74)	*U* =307,977	0.701	*r* = 0.008
BMI, *M* (*SD*)^*^	23.1 (4.27)	23.5 (4.26)	*U =* 318,402	0.105	*r =* 0.032
Relationship status, *n* (%)			*χ^2^*(3, 2569) = 3.66	0.300	φ_c_ = 0.038
-Married	99 (4.31)	10 (3.68)			
-In a committed relationship	968 (42.14)	118 (43.38)			
-Single	1,226 (53.37)	142 (52.21)			
-Divorced	4 (0.17)	2 (0.74)			
Residential status, *n* (%)			*χ^2^* (1, 2569) = 2.51	0.113	φ = 0.031
-Alone	614 (26.73)	60 (22.06)			
-With partner/spouse/children/others	1,683 (73.27)	212 (77.94)			
Migration history, *n* (%)	326 (14.19)	30 (11.03)	*χ^2^* (1, 2569) = 1.78	0.182	φ = 0.026
Being a parent, *n* (%)	88 (3.83)	6 (2.21)	*χ^2^*(1, 2569) = 1.39	0.238	φ = 0.023

^*^Sample size *n*= 2,257 and *n*= 266 respectively as outliers were excluded from the analysis.

### 3.2 Mental health outcomes

Students who reported experiencing discrimination based on sexual orientation showed significantly higher rates of diagnosed mental illness compared to those without such experiences (55.15% vs. 31.48%, χ^2^ (1, 2569) = 59.61, *p* < .001, φ =.153) (see Table 3). Consistent with this, they also scored higher on measures of depressive symptoms (PHQ-9: *M* = 12.2, *SD* = 5.82 vs. *M* = 9.68, *SD* = 5.65; *U* = 352626, *p* < .001, *r* =.132) and generalized anxiety (GAD-7: *M* = 11.3, *SD* = 5.06 vs. *M* = 9.19, *SD* = 5.05; *U* = 386695.5, *p* < .001, *r* =.127). Furthermore, suicidal thoughts were reported more frequently by students facing discrimination based on sexual orientation (41.54%) than by those not reporting this kind of discrimination (23.34%, χ^2^ (1, 2569) = 42.655, *p* < .001, φ =.139). In contrast, no significant differences were found between groups in alcohol use as measured by the AUDIT-C (*U* = 277323.5, *p* =.856, *r* =.004) or in eating disorder symptomatology assessed by the EDE-Q8 (*U* = 321692, *p* =.116, *r* =.031).

**Table 3:** Mental health outcomes, financial status and impairment status of student samples (*N* = 2,569).

Variable	Students without discrimination based on sexual orientation (*n* = 2,297)	Students with discrimination based on sexual orientation (*n* = 272)	Test	*p*	Effect size
Diagnosed with mental illness, *n* (%)	721 (31.48)	150 (55.15)	*χ^2^*(1, 2569) = 59.61	< 0.001	φ = 0.153
PHQ-9, *M* (*SD*)	9.68 (5.65)	12.2 (5.82)	*U* = 352,626	< 0.001	*r =* 0.132
Suicidal thoughts (PHQ-9 item 9), *n* (%)	535 (23.34)	113 (41.54)	*χ^2^* (1, 2569) = 42.655	< 0.001	φ = 0.139
GAD-7, *M (SD)*	9.19 (5.05)	11.3 (5.06)	*U* = 386,695.5	< 0.001	*r* = 0.127
UCLA-3, *M (SD)*	5.75 (1.87)	6.01 (1.79)	*U =* 30,2261.5	0.018	*r* = 0.047
AUDIT-C, *M (SD)*	2.33 (2.13)	2.40 (2.26)	*U=* 277,323	0.856	*r =* 0.004
EDE-Q8, *M (SD)*	1.73 (1.57)	1.83 (1.52)	*U =* 321,692	0.116	*r =* 0.031
Income, n (%)			*χ^2^* (7, 2569) = 11.3	0.957	*φ_*c*_ =* 0.066
No income	218 (9.49)	20 (7.35)			
0–499 €	300 (13.1)	32 (11.8)			
500–999 €	917 (39.9)	108 (39.7)			
1,000–1,499 €	627 (27.3)	83 (30.5)			
1,500–1,999 €	116 (5.05)	13 (4.78)			
2,000–2,999 €	58 (2.53)	7 (2.57)			
3,000 and more	16 (0.7)	1 (0.37)			
No answer	45 (1.96)	8 (2.94)			
Students with impairment, *n* (%)	273 (11.9)	57 (21)	*χ^2^* (1, 2569) = 30.294	< 0.001	φ = 0.107
Impairment type			*χ^2^* (8, 330) = 7.752	0.458	*φ_*c*_* = 0.153
-Psychological disorder	122 (5.31)	24 (8.82)			
-Physical chronic illness	35 (1.52)	4 (1.47)			
-Learning disability	9 (0.35)	1 (0.3)			
-Mobility impairment	1 (0.04)	0 (0)			
-Visual impairment or blindness	2 (0.09)	2 (0.74)			
-Hearing impairment or deafness	1 (0.04)	0 (0)			
-Equally severe multiple impairments	34 (1.48)	6 (2.21)			
-Other impairment or illness	67 (2.92)	20 (7.35)			
-No information	2 (0.09)	0 (0)			
Help seeking behavior *n*, (%)					
-Psychiatrist	361 (16.4)	88 (33.8)	*χ^2^* (1, 2569) = 46.392	< 0.001	φ = 0.137
-Psychotherapy	771 (35)	152 (58.5)	*χ^2^* (1, 2569) = 53.645	< 0.001	φ = 0.148
-Psychological/psychosocial counseling	589 (26.7)	108 (41.5)	*χ^2^* (1, 2569) = 24.388	< 0.001	φ = 0.100
-Alternative/non-medical practitioner	105 (4.77)	30 (11.5)	*χ^2^* (1, 2569) = 19.301	< 0.001	φ = 0.089
-Stay in a psychiatric/psychosomatic clinic	187 (8.49)	39 (15)	*χ^2^* (1, 2569) = 11.063	< 0.001	φ = 0.067
-Online help services for mental health problems (e.g., Apps, self-management programs)	497 (22.6)	93 (35.8)	*χ^2^* (1, 2569) = 21.555	< 0.001	φ = 0.094
-Self-help books/guides	670 (30.4)	98 (37.7)	*χ^2^* (1, 2569) = 5.408	0.020	φ = 0.047

### 3.3 Loneliness

Based on the UCLA 3-Item Loneliness Scale (UCLA-3), students who experienced discrimination based on their sexual orientation did not report significantly higher levels of loneliness than those without such experiences (M = 6.01, SD = 1.79 vs. M = 5.75, SD = 1.87; U = 302261.5, p = 0.018, r = 0.047).

### 3.4 Impairment status

A greater proportion of students reporting discrimination based on sexual orientation indicated having some form of impairment compared to those not reporting such discrimination (21% vs. 11.9%, χ^2^ (1, 2569) = 30.294, *p* < .001, φ =.107). However, the distribution of different impairment types, including psychological disorders, physical chronic illnesses, learning disabilities, mobility impairments, sensory impairments, multiple severe impairments, and other types, did not significantly differ between groups (χ^2^ (8, 330) = 7.752, *p* =.458, φ_*c*_ = 0.153).

### 3.5 Income

Monthly income distribution did not differ significantly between students with and without discrimination experiences based on sexual orientation (χ^2^ (7, 2569) = 11.3, *p* =.957, φ_*c*_ =.066). Income categories ranged broadly across both groups, with the largest proportion of students falling within the 500–999 € per month range.

### 3.6 Help-seeking behavior

University students reporting discrimination based on sexual orientation (*n* = 272) demonstrated higher rates of help-seeking behavior across six out of seven assessed domains compared to students without such experiences (*n* = 2,297). Specifically, 33.8% of students with discrimination sought help from psychiatrists compared to 16.4% without discrimination (χ^2^ (1, 2569) = 46.392, *p* < .001, φ = 0.137). Psychotherapy was utilized by 58.5% vs. 35% (χ^2^ (1, 2569) = 53.645, *p* < .001, φ = 0.148), and psychological/psychosocial counseling by 41.5% vs. 26.7% (χ^2^ (1, 2569) = 24.388, *p* < 0.001, φ = 0.100). Use of alternative/non-medical practitioners was reported by 11.5% vs. 4.77% (χ^2^ (1, 2569) = 19.301, *p* < .001, φ = 0.089). Additionally, 15% of students with discrimination had stays in psychiatric or psychosomatic clinics compared to 8.49% without discrimination (χ^2^ (1, 2569) = 11.063, *p* < 0.001, φ = 0.067). Online help services such as apps and self-management programs were accessed by 35.8% vs. 22.6% (χ^2^ (1, 2569) = 21.555, *p* < 0.001, φ = 0.094). There was no statistically significant difference in the use of self-help books or guides by 37.7% vs. 30.4% (χ^2^ (1, 2569) = 5.408, *p* = 0.020, φ = 0.047) (see Table 3).

## 4 Discussion

**Figure 1:**
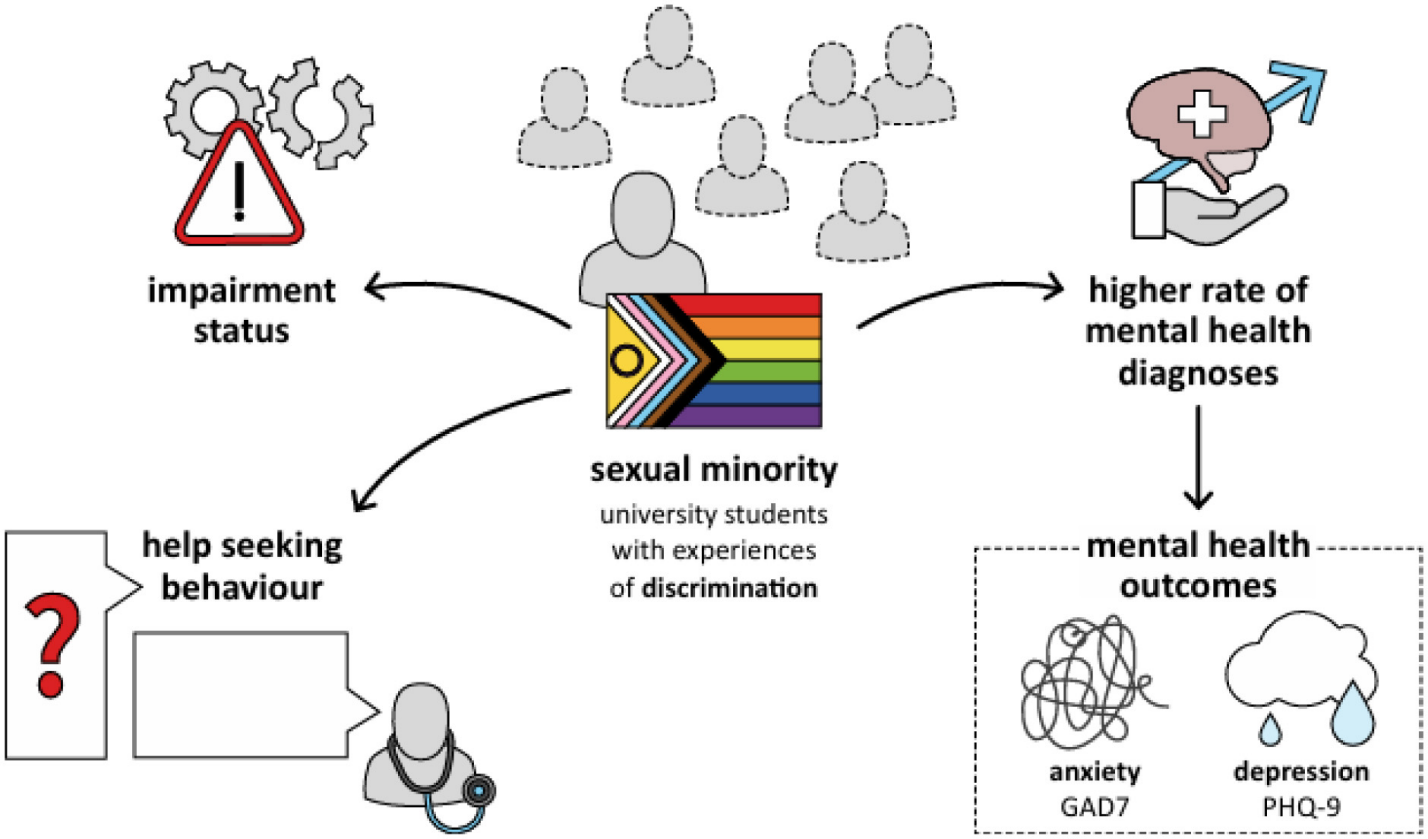
Illustration of the main findings.

Our study's findings demonstrate that university students who experienced discrimination based on sexual orientation faced significantly greater mental health challenges compared to their peers that did not experience discrimination based on sexual orientation (see Figure 1). These students reported higher rates of diagnosed mental illness, elevated symptoms of depression and generalized anxiety, and an increased prevalence of suicidal thoughts. No significant differences were found regarding alcohol use, eating disorder symptoms, or loneliness. Furthermore, a larger proportion of these students reported having some form of impairment that made studying at university more difficult. However, the types of impairment did not differ between groups. Income distribution was similar across groups. Notably, students facing discrimination exhibited consistently higher rates of help-seeking behavior across a wide range of support modalities.

Overview of the main findings of this study illustrating how sexual minority students are potentially impacted by a higher rate of mental health diagnoses, increased help-seeking behavior, impairments and mental health symptoms such as anxiety and depression.

### 4.1 Mental health outcomes and loneliness

The results of our study showed that students who experienced discrimination based on their sexual orientation reported worse mental health outcomes. Specifically, their scores for depression (PHQ-9) and anxiety (GAD-7) were higher than those of students who did not experience such discrimination. Our results are consistent with a large body of literature highlighting that sexual minority people around the world face higher rates of mental illness compared to cisgender heterosexuals (34–38), mainly because of the physical and psychological stress caused by being part of a minority and its associated stigma (20, 39).

Scores for alcohol use (AUDIT-C) and eating disorders (EDE-Q8) were surprisingly similar between both groups. One plausible explanation for the lack of difference in alcohol use is the long-term impact of the COVID-19 pandemic on young adults, which likely led to an initial reduction in alcohol consumption (40–42), followed by a rebound increase in alcohol consumption (41) that may have diminished or masked any group-specific patterns. Unexpectedly, we did not find a difference in mean EDE-Q8 scores between the groups, even though several studies reported a higher prevalence of eating disorders reported among young sexual minority individuals (43–45). This null finding may reflect the brief nature of the EDE-Q8 self-report instrument (30), which might be less sensitive than clinical interviews or longer eating disorder inventories for detecting subtle symptoms in a non-clinical student sample.

An important factor to consider regarding this result is the gender composition of the samples. The group facing discrimination included significantly fewer female participants compared to the non-discriminated group (59.46% vs. 71.75%, see Table 2). Given that eating disorders are more common among women (46, 47), this imbalance may have influenced our results and may have contributed to the absence of group differences in disordered eating behaviors.

In our study, there was no significant difference in loneliness (UCLA-3) between students who had experienced discrimination based on sexual orientation and those who had not. Although previous studies have reported higher levels of loneliness among sexual minority individuals (21, 48), this association was not statistically confirmed in our sample. This null-finding may be partly due to the use of a brief three-item measure of loneliness (29), which could have limited the sensitivity of the assessment. Furthermore, supportive social networks between students established within the university context might help protect students from experiencing loneliness (49). Additionally, a German translation of the UCLA 3-Item Loneliness scale was used. The German translation of the UCLA-3 showed high internal consistency with Cronbach's α of 0.80 (50).

### 4.2 Impairment status

Furthermore, a higher proportion of students who reported discrimination based on sexual orientation indicated having some form of impairment compared to those without experiences of discrimination, although the types of impairments were similarly distributed between the groups. This suggests that discrimination based on sexual orientation may be associated with an impairment that makes studying at university more difficult and also restricts participation in academic life. In addition, research suggests that sexual minorities may encounter challenges stemming from prejudice and discrimination, which can negatively affect their relationships, overall social wellbeing (51), and also lead to reduced participation in the labor market (52).

### 4.3 Income differences

There were no significant differences in monthly income between students with and without experiences of discrimination in our study, with both groups showing a similar distribution across income categories. The lack of family support commonly reported among young sexual minority adults (53, 54) is therefore more likely to impact psychosocial wellbeing than financial support. However, further longitudinal research with larger and more diverse samples, beyond the student population, as well as longitudinal studies, are needed to draw more definitive conclusions. It is noteworthy that in both groups, 218 individuals (9.49%) and 20 individuals (7.35%), respectively, reported having no own income source. However, it is common among university students to have no personal income and instead receive financial support from family members, for example.

### 4.4 Help-seeking behavior

In our study we found higher help-seeking behavior across six out of seven help-seeking modalities among students that experienced discrimination based on sexual orientation. This is in line with other current research results (55, 56), underscoring the urgent need for mental health professionals to be well-prepared to address the specific psychosocial issues stemming from discrimination and stigma related to sexual orientation. Mental health professionals, particularly psychiatrists and psychotherapists, should therefore receive specialized training to recognize and respond to the unique psychosocial impacts of discrimination related to sexual orientation, for example LGBTQ-affirmative cognitive-behavioral therapy (57). Additionally, psychosocial counseling services at universities play a critical role in supporting affected students. It is essential that these services understand and are sensitive to the distinct challenges faced by students experiencing such discrimination. By fostering an inclusive, open, and supportive environment, counseling services can ensure that all students, especially those who have encountered discrimination, feel welcomed, understood, and encouraged to seek the necessary support for their mental wellbeing. However, there was no statistically significant difference in the use of self-help books or guides between students with and without experiences of discrimination based on sexual orientation. However, such resources may reflect a more general form of help-seeking behavior that is also present among all university student groups, regardless of experiences of discrimination based on sexual orientation.

## 5 Limitations

A key limitation of this study is that sexual orientation data were not collected. Instead, participants only indicated whether they had experienced discrimination based on their sexual orientation. Consequently, the pronounced effects observed in the study may partly reflect an unclear differentiation of how many students with various sexual minority identities were actually subject to discrimination. Furthermore, it is possible that discriminatory experiences reported in this study also included those of heterosexual individuals, as discrimination due to sexual orientation was assessed without recording participants' sexual orientation. However, the survey question regarding discrimination based on sexual orientation explicitly named examples such as “gay” or “lesbian” rendering it highly unlikely that heterosexual participants felt addressed or reported such experiences. Consistent with the minority stress theory proposed by Meyer et al. (20), we focused on perceived discrimination based on sexual orientation as the primary exposure rather than on sexual identity labels, in order to underscore the specific impact of discriminatory experiences on mental health. Nevertheless, future research would benefit from distinguishing between the diverse sexual orientations and identities within the queer community to better understand the specific experiences and impacts of discrimination across different groups and their intersections. Additionally, the study did not assess the frequency, duration, or severity of discrimination experiences, all of which could influence mental health outcomes (58).

Moreover, due to the cross-sectional design of the study, causal relationships cannot be established. Mental health outcomes were measured using screening tools rather than clinical diagnoses. While self-reported data may be subject to recall bias or social desirability bias, it simultaneously offers the advantage of providing a broad overview of the student cohort's experiences and mental health status.

## 6 Conclusion

Overall, this paper highlights that discrimination based on sexual orientation significantly compromises mental health among university students, demonstrating that both intensified research and mental health service provision are necessary to foster equity, resilience, and better outcomes for young sexual minority populations.

For further research, it is essential to expand beyond cross-sectional designs and limited samples, incorporating longitudinal approaches and more diverse, intersectional populations to deepen our understanding of how discrimination affects mental health over time and across different distinct queer identities.

Mental health professionals should receive targeted training in LGBTQ-affirmative care to effectively recognize and respond to the psychosocial impacts of discrimination and stigma due to sexual orientation. University counseling services should create inclusive and supportive environments that encourage help-seeking and promote psychological wellbeing among sexual minority students with experiences of discrimination.

## Data Availability

The raw data supporting the conclusions of this article will be made available by the authors, without undue reservation.

## References

[1] Lund EM Burgess CM. Sexual and gender minority health care disparities: barriers to care and strategies to bridge the gap. Prim Care. (2021) 48:179–89. doi: 10.1016/j.pop.2021.02.007 33985698

[2] Bilimoria D Stewart AJ. “Don't ask, don't tell”: the academic climate for lesbian, gay, bisexual, and transgender faculty in science and engineering. NWSA J. (2009) 21:85–103. doi: 10.1353/ff.2009.a316151

[3] Mansh M Garcia G Lunn MR. From patients to providers: changing the culture in medicine toward sexual and gender minorities. Acad Med. (2015) 90:574–80. doi: 10.1097/ACM.0000000000000656 25650825

[4] Przedworski JM VanKim NA Eisenberg ME McAlpine DD Lust KA Laska MN. Self-reported mental disorders and distress by sexual orientation: results of the Minnesota college student health survey. Am J Prev Med. (2015) 49:29–40. doi: 10.1016/j.amepre.2015.01.024 25997903 PMC4476922

[5] Pagliaccio D. Mental health disparities among sexual and gender minority students in higher education. J Am Coll Health. (2024) 73:1–12. doi: 10.1080/07448481.2024.2404944 39533453

[6] Grant JE Odlaug BL Derbyshire K Schreiber LRN Lust K Christenson G. Mental health and clinical correlates in lesbian, gay, bisexual, and queer young adults. J Am Coll Health. (2014) 62:75–8. doi: 10.1080/07448481.2013.844697 24313699

[7] Reed E Prado G Matsumoto A Amaro H. Alcohol and drug use and related consequences among gay, lesbian and bisexual college students: role of experiencing violence, feeling safe on campus, and perceived stress. Addict Behav. (2010) 35:168–71. doi: 10.1016/j.addbeh.2009.09.005 19796880 PMC2783782

[8] Marshal MP Dietz LJ Friedman MS Stall R Smith HA McGinley J . Suicidality and depression disparities between sexual minority and heterosexual youth: a meta-analytic review. J Adolesc Health. (2011) 49:115–23. doi: 10.1016/j.jadohealth.2011.02.005 21783042 PMC3649127

[9] Lytle MC Silenzio VMB Homan CM Schneider P Caine ED. Suicidal and help-seeking behaviors among youth in an online lesbian, gay, bisexual, transgender, queer, and questioning social network. J Homosex. (2018) 65:1916–33. doi: 10.1080/00918369.2017.1391552 29020574

[10] Gulec H Torun T Da Prado AS Bauer S Rummel-Kluge C Kohls E. Eating attitudes and depressive symptoms in a LGBTIQ sample in Turkey. Front Psychiatry. (2022) 13:1014253. doi: 10.3389/fpsyt.2022.1014253 36440428 PMC9691651

[11] Herek GM Kimmel DC Amaro H Melton GB. Avoiding heterosexist bias in psychological research. Am Psychol. (1991) 46:957–63. doi: 10.1037/0003-066X.46.9.957 1958014

[12] Gallup. LGBTQ+ Identification in U.S. Now at 7.6% (2024). Available online at: https://news.gallup.com/poll/611864/lgbtq-identification.aspx (Accessed August 3, 2025).

[13] Ryan C Huebner D Diaz RM Sanchez J. Family rejection as a predictor of negative health outcomes in white and Latino lesbian, gay, and bisexual young adults. Pediatrics. (2009) 123:346–52. doi: 10.1542/peds.2007-3524 19117902

[14] Al-Ajlouni YA Park SH Safren SA Kreski NT Elbel B Trinidad A . High financial hardship and mental health burden among gay, bisexual and other men who have sex with men. J Gay Lesbian Ment Health. (2020) 24:308–21. doi: 10.1080/19359705.2019.1688217 32884610 PMC7462116

[15] Garvey JC Jackson R Dolan CV Simpfenderfer AD. The queer price of college: mapping the financial landscape for queer students. J Coll Stud Dev. (2022) 63:449–54. doi: 10.1353/csd.2022.0037

[16] Dunbar MS Sontag-Padilla L Ramchand R Seelam R Stein BD. Mental health service utilization among lesbian, gay, bisexual, and questioning or queer college students. J Adolesc Health. (2017) 61:294–301. doi: 10.1016/j.jadohealth.2017.03.008 28549595

[17] DiPlacido J Fallahi CR. Stigma and sexual and gender minority mental health. In:Rothblum ED, editor. The Oxford Handbook of Sexual and Gender Minority Mental Health. Oxford University Press (2020). p. 418–28. doi: 10.1093/oxfordhb/9780190067991.013.37

[18] Martos AJ Wilson PA Gordon AR Lightfoot M Meyer IH. “Like finding a unicorn”: healthcare preferences among lesbian, gay, and bisexual people in the United States. Soc Sci Med. (2018) 208:126–33. doi: 10.1016/j.socscimed.2018.05.020 29803970 PMC6382457

[19] Hoffman ND Freeman K Swann S. Healthcare preferences of lesbian, gay, bisexual, transgender and questioning youth. J Adolesc Health. (2009) 45:222–9. doi: 10.1016/j.jadohealth.2009.01.009 19699417 PMC2773204

[20] Meyer IH Frost DM. Minority stress and the health of sexual minorities. In:Patterson CJ D'Augelli AR, editors. Handbook of Psychology and Sexual Orientation. Oxford University Press (2012). p. 252–66. doi: 10.1093/acprof:oso/9780199765218.003.0018

[21] Eres R Postolovski N Thielking M Lim MH. Loneliness, mental health, and social health indicators in LGBTQIA+ Australians. Am J Orthopsychiatry. (2021) 91:358–66. doi: 10.1037/ort0000531 33315419

[22] Mushtaq R Shoib S Shah T Mushtaq S. Relationship between loneliness, psychiatric disorders and physical health? A review on the psychological aspects of loneliness. J Clin Diagn Res. (2014) 8:WE01–4. doi: 10.7860/JCDR/2014/10077.4828 25386507 PMC4225959

[23] Mereish EH Poteat VP. A relational model of sexual minority mental and physical health: the negative effects of shame on relationships, loneliness, and health. J Couns Psychol. (2015) 62:425–37. doi: 10.1037/cou0000088 26010289 PMC4501878

[24] Lee JH Gamarel KE Bryant KJ Zaller ND Operario D. Discrimination, mental health, and substance use disorders among sexual minority populations. LGBT Health. (2016) 3:258–65. doi: 10.1089/lgbt.2015.0135 27383512 PMC4976222

[25] Plöderl M Tremblay P. Mental health of sexual minorities. A systematic review. Int Rev Psychiatry. (2015) 27:367–85. doi: 10.3109/09540261.2015.1083949 26552495

[26] Kroenke K Spitzer RL Williams JB. The PHQ-9: validity of a brief depression severity measure. J Gen Intern Med. (2001) 16:606–13. doi: 10.1046/j.1525-1497.2001.016009606.x 11556941 PMC1495268

[27] Spitzer RL Kroenke K Williams JBW Löwe B. A brief measure for assessing generalized anxiety disorder: the GAD-7. Arch Intern Med. (2006) 166:1092–7. doi: 10.1001/archinte.166.10.1092 16717171

[28] Bradley KA DeBenedetti AF Volk RJ Williams EC Frank D Kivlahan DR. AUDIT-C as a brief screen for alcohol misuse in primary care. Alcohol Clin Exp Res. (2007) 31:1208–17. doi: 10.1111/j.1530-0277.2007.00403.x 17451397

[29] Hughes ME Waite LJ Hawkley LC Cacioppo JT. A short scale for measuring loneliness in large surveys: results from two population-based studies. Res Aging. (2004) 26:655–72. doi: 10.1177/0164027504268574 18504506 PMC2394670

[30] Kliem S Mößle T Zenger M Strauß B Brähler E Hilbert A. The eating disorder examination-questionnaire 8: a brief measure of eating disorder psychopathology (EDE-Q8). Int J Eat Disord. (2016) 49:613–6. doi: 10.1002/eat.22487 26711183

[31] Mahon CP Lombard-Vance R Kiernan G Pachankis JE Gallagher P. Social anxiety among sexual minority individuals: a systematic review. Psychol Sex. (2022) 13:818–62. doi: 10.1080/19419899.2021.1936140

[32] Santoniccolo F Rollè L. The role of minority stress in disordered eating: a systematic review of the literature. Eat Weight Disord. (2024) 29:41. doi: 10.1007/s40519-024-01671-7 38850334 PMC11162380

[33] Cohen J. Statistical Power Analysis for the Behavioral Sciences. New York: Routledge (2013). doi: 10.4324/9780203771587

[34] King M Semlyen J Tai SS Killaspy H Osborn D Popelyuk D . A systematic review of mental disorder, suicide, and deliberate self harm in lesbian, gay and bisexual people. BMC Psychiatry. (2008) 8:70. doi: 10.1186/1471-244X-8-70 18706118 PMC2533652

[35] Tan KKH Saw ATW. Prevalence and correlates of mental health difficulties amongst LGBTQ people in Southeast Asia: a systematic review. J Gay Lesbian Ment Health. (2023) 27:401–20. doi: 10.1080/19359705.2022.2089427

[36] Hayek SE Kassir G Cherro M Mourad M Soueidy M Zrour C . Mental health of LGBTQ individuals who are Arab or of an Arab Descent: a systematic review. J Homosex. (2023) 70:2439–61. doi: 10.1080/00918369.2022.2060624 35499284

[37] Steele LS Daley A Curling D Gibson MF Green DC Williams CC . LGBT identity, untreated depression, and unmet need for mental health services by sexual minority women and trans-identified people. J Womens Health. (2017) 26:116–27. doi: 10.1089/jwh.2015.5677 27898255

[38] Borgogna NC McDermott RC Aita SL Kridel MM. Anxiety and depression across gender and sexual minorities: Implications for transgender, gender nonconforming, pansexual, demisexual, asexual, queer, and questioning individuals. Psychol Sex Orientat Gend Divers. (2019) 6:54–63. doi: 10.1037/sgd0000306

[39] Hatzenbuehler ML Pachankis JE. Stigma and minority stress as social determinants of health among lesbian, gay, bisexual, and transgender youth: research evidence and clinical implications. Pediatr Clin North Am. (2016) 63:985–97. doi: 10.1016/j.pcl.2016.07.003 27865340

[40] Vera BDV Carmona-Márquez J Lozano-Rojas ÓM Parrado-González A Vidal-Giné C Pautassi RM . Changes in alcohol use during the COVID-19 pandemic among young adults: the prospective effect of anxiety and depression. J Clin Med. (2021) 10. doi: 10.3390/jcm10194468 34640485 PMC8509511

[41] Gohari MR Varatharajan T MacKillop J Leatherdale ST. Examining the impact of the COVID-19 pandemic on youth alcohol consumption: longitudinal changes from pre-to intra-pandemic drinking in the COMPASS study. J Adolesc Health. (2022) 71:665–72. doi: 10.1016/j.jadohealth.2022.07.007 36088229 PMC9451939

[42] Ryerson NC Wilson OWA Pena A Duffy M Bopp M. What happens when the party moves home? The effect of the COVID-19 pandemic on US college student alcohol consumption as a function of legal drinking status using longitudinal data. Transl Behav Med. (2021) 11:772–4. doi: 10.1093/tbm/ibab006 PMC7928597 33598696

[43] McClain Z Peebles R. Body image and eating disorders among lesbian, gay, bisexual, and transgender youth. Pediatr Clin North Am. (2016) 63:1079–90. doi: 10.1016/j.pcl.2016.07.008 27865334 PMC6402566

[44] Calzo JP Blashill AJ Brown TA Argenal RL. Eating disorders and disordered weight and shape control behaviors in sexual minority populations. Curr Psychiatry Rep. (2017) 19:49. doi: 10.1007/s11920-017-0801-y 28660475 PMC5555626

[45] Arikawa AY Ross J Wright L Elmore M Gonzalez AM Wallace TC. Results of an online survey about food insecurity and eating disorder behaviors administered to a volunteer sample of self-described LGBTQ+ young adults aged 18 to 35 years. J Acad Nutr Diet. (2021) 121:1231–41. doi: 10.1016/j.jand.2020.09.032 33158800

[46] Murray SB. Gender identity and eating disorders: the need to delineate novel pathways for eating disorder symptomatology. J Adolesc Health. (2017) 60:1–2. doi: 10.1016/j.jadohealth.2016.10.004 27838236

[47] Smink FRE van Hoeken D Hoek HW. Epidemiology of eating disorders: incidence, prevalence and mortality rates. Curr Psychiatry Rep. (2012) 14:406–14. doi: 10.1007/s11920-012-0282-y 22644309 PMC3409365

[48] Elmer EM van Tilburg T Fokkema T. Minority stress and loneliness in a global sample of sexual minority adults: the roles of social anxiety, social inhibition, and community involvement. Arch Sex Behav. (2022) 51:2269–98. doi: 10.1007/s10508-021-02132-3 35084615 PMC9192366

[49] Wenig V Heinrichs K Heumann E Lehnchen J Burian J Deptolla Z . Social-ecological factors associated with loneliness in university students: results from the German cross-sectional StudiBiFra study. Front Psychiatry. (2025) 16:1469811. doi: 10.3389/fpsyt.2025.1469811 40692650 PMC12277272

[50] da Silva Prado A Kohls E Hug J Endres KJ Rummel-Kluge C. University students' mental health in 2025: generation overwhelmed?. Res Square. doi: 10.21203/rs.3.rs-8561593/v1

[51] Doyle DM Molix L. Disparities in social health by sexual orientation and the etiologic role of self-reported discrimination. Arch Sex Behav. (2016) 45:1317–27. doi: 10.1007/s10508-015-0639-5 26566900 PMC4866902

[52] Bränström R Narusyte J Svedberg P. Sexual-orientation differences in risk of health-related impaired ability to work and to remain in the paid workforce: a prospective population-based twin study. BMC Public Health. (2023) 23:454. doi: 10.1186/s12889-023-15384-6 36890524 PMC9996859

[53] McConnell EA Birkett M Mustanski B. Families matter: social support and mental health trajectories among lesbian, gay, bisexual, and transgender youth. J Adolesc Health. (2016) 59:674–80. doi: 10.1016/j.jadohealth.2016.07.026 27707515 PMC5217458

[54] Shilo G Savaya R. Effects of family and friend support on LGB youths' mental health and sexual orientation milestones. Fam Relat. (2011) 60:318–30. doi: 10.1111/j.1741-3729.2011.00648.x

[55] Baams L Luca SM de Brownson C. Use of mental health services among college students by sexual orientation. LGBT Health. (2018) 5:421–30. doi: 10.1089/lgbt.2017.0225 30280997 PMC6207152

[56] Chang AR Rastogi R Woolverton GA Chen JA Stevens C Reisner SL . Mental health help-seeking willingness among US college students: a resilience factor associated with many sexual minority identities. Psychiatry Res. (2024) 342:116173. doi: 10.1016/j.psychres.2024.116173 39307108 PMC11617266

[57] Pachankis JE Soulliard ZA Seager vDI Layland EK Clark KA Levine DS . Training in LGBTQ-affirmative cognitive behavioral therapy: a randomized controlled trial across LGBTQ community centers. J Consult Clin Psychol. (2022) 90:582–99. doi: 10.1037/ccp0000745 35901370 PMC9434976

[58] Lei Y Shah V Biely C Jackson N Dudovitz R Barnert E . Discrimination and subsequent mental health, substance use, and well-being in young adults. Pediatrics. (2021) 148:e2021051378. doi: 10.1542/peds.2021-051378 34816276 PMC9126825

[59] Martin A Rief W Klaiberg A Braehler E. Validity of the Brief Patient Health Questionnaire Mood Scale (PHQ-9) in the general population. Gen Hosp Psychiatry. (2006) 28:71–7. doi: 10.1016/j.genhosppsych.2005.07.003 16377369

[60] Löwe B Decker O Müller S Brähler E Schellberg D Herzog W . Validation and standardization of the Generalized Anxiety Disorder Screener (GAD-7) in the general population. Med Care. (2008) 46:266–74. doi: 10.1097/MLR.0b013e318160d093 18388841

[61] Bush K Kivlahan DR McDonell MB Fihn SD Bradley KA. The AUDIT alcohol consumption questions (AUDIT-C): an effective brief screening test for problem drinking. Ambulatory Care Quality Improvement Project (ACQUIP). Alcohol Use Disorders Identification Test. Arch Intern Med. (1998) 158:1789–95. doi: 10.1001/archinte.158.16.1789 9738608

[62] Rumpf H-J Hapke U Meyer C John U. Screening for alcohol use disorders and at-risk drinking in the general population: psychometric performance of three questionnaires. Alcohol Alcohol. (2002) 37:261–8. doi: 10.1093/alcalc/37.3.261 12003915

[63] Richter F Strauß B Berger U. Deutschsprachige Kurzskalen zur Erfassung auffälligen Essverhaltens. Psychother Psychosom Med Psychol. (2018) 68:99–108. doi: 10.1055/s-0043-106433 28718871

